# DSM-5 insomnia disorder in pregnancy: associations with depression, suicidal ideation, and cognitive and somatic arousal, and identifying clinical cutoffs for detection

**DOI:** 10.1093/sleepadvances/zpac006

**Published:** 2022-03-11

**Authors:** David A Kalmbach, Philip Cheng, Andrea Roth, Thomas Roth, Leslie M Swanson, Louise M O’Brien, David M Fresco, Nicholas C Harb, Andrea S Cuamatzi-Castelan, Anthony N Reffi, Christopher L Drake

**Affiliations:** 1 Thomas Roth Sleep Disorders & Research Center, Henry Ford Health System, Detroit, MIUSA; 2 Department of Pulmonary & Critical Care and Sleep Medicine, Wayne State University School of Medicine, Detroit, MIUSA; 3 Pediatric Sleep Medicine, Thriving Minds Behavioral Health, Livonia, MIUSA; 4 Department of Psychiatry, University of Michigan, Ann Arbor, MIUSA; 5 Departments of Obstetrics & Gynecology and Neurology, University of Michigan, Ann Arbor, MIUSA

**Keywords:** ICSD-3, perinatal, Presleep Arousal Scale, Insomnia Severity Index, Pittsburgh Sleep Quality Index, Epworth Sleepiness Scale, daytime sleepiness

## Abstract

**Study Objectives:**

The study had three primary goals. First, we estimated survey-assessed DSM-5 insomnia disorder rates in pregnancy, and described associated sociodemographics, and sleep-wake and mental health symptoms. Second, we derived cutoffs for detecting DSM-5 insomnia disorder using common self-report measures of sleep symptoms. Third, we identified clinically relevant cut-points on measures of nocturnal cognitive and somatic arousal.

**Methods:**

Ninety-nine women (85.9% in the 2^nd^ trimester) completed online surveys including DSM-5 insomnia disorder criteria, the Insomnia Severity Index (ISI), Pittsburgh Sleep Quality Index (PSQI), Presleep Arousal Scale’s Cognitive (PSASC) and Somatic (PSASS) factors, and Edinburgh Postnatal Depression Scale.

**Results:**

DSM-5 insomnia disorder rate was 19.2%. Insomnia was associated with depression, suicidality, nocturnal cognitive and somatic arousal, and daytime sleepiness. An ISI scoring method that aligns with DSM-5 criteria yielded excellent metrics for detecting insomnia disorder and good sleep. Regarding quantitative cutoffs, ISI ≥ 10 and ISI ≥ 11 (but not ISI ≥ 15) were supported for detecting DSM-5 insomnia, whereas ISI ≤ 7 and ISI ≤ 9 performed well for detecting good sleep. PSQI cutoff of 5 was supported for detecting insomnia and good sleep. The optimal cutoff for nocturnal cognitive arousal was PSASC ≥ 18, whereas the optimal cutoff for somatic arousal was PSASS ≥ 13.

**Conclusions:**

Insomnia disorder affects a large segment of pregnant women. Empirically derived cutoffs for insomnia, good sleep, cognitive arousal, and somatic arousal may inform case identification and future perinatal sleep research methodology.

**Statement of Significance**

A large segment of the pregnant population endorses insomnia symptoms. In the present study of pregnant women mostly in the 2^nd^ trimester, we estimated that 19.2% meet DSM-5 criteria for insomnia disorder. We showed that DSM-5 insomnia disorder corresponds to elevated rates of short sleep duration, depression, suicidal ideation, excessive daytime sleepiness, and nocturnal cognitive and somatic hyperarousal. Interestingly, cognitive and somatic hyperarousal were linked to different sleep complaints in insomnia. Nocturnal cognitive arousal mapped onto difficulties falling asleep and wakefulness in the middle of the night, whereas somatic arousal was implicated in early morning awakenings. Empirically derived cutoffs for insomnia disorder and nighttime arousal may guide future research methodology of sleep research in this patient population.

## Introduction

Insomnia during pregnancy is a recently recognized public health emergency. Burgeoning interest in perinatal sleep has resulted in a proliferation of studies examining insomnia in pregnancy over the past 15 years. Recent meta-analytic estimates suggest that 25–27% of pregnant women in the first two trimesters endorse clinically important insomnia symptoms, and that this rate increases to about 40% in the third trimester [1–3]. Several large-scale studies have shown that over half of women experience insomnia by the end of pregnancy [4–9]. Women who develop prenatal insomnia report reduced quality of life [10,11], and are at increased risk for preterm birth [12], maternal depression [8,13–15], suicidal ideation [16], postpartum pain [17], and impaired mother-to-infant bonding [18,19]. Although the literature offers clear evidence that prenatal insomnia corresponds to adverse outcomes for mothers and their children, the field has been hampered by an important methodological limitation: classification methods for identifying cases of prenatal insomnia have been highly variable [1]. Even when investigations on prenatal insomnia use the same assessment measure, the selected cutoffs used to define cases with insomnia vary widely across studies [1]. Accurate case identification is important for producing valid and reliable estimates of rates, descriptions of morbidity, and tracking the effectiveness of interventions.

Variations in classifying cases with insomnia are partly due to ‘insomnia’ serving as an umbrella term reflecting both insomnia disorder and a variety of symptom presentations (i.e., symptoms of clinical relevance that do not meet diagnostic criteria). The vast majority of research in prenatal insomnia has focused on clinically significant insomnia severity [1,2], which inevitably includes both women with elevated symptoms and the disorder. While this research has greatly advanced our knowledge of sleep complaints in pregnancy, a resultant gap is that we know relatively little about the rates and morbidity of insomnia disorder in pregnancy.

However, a recent study differentiated rates of DSM-5 insomnia disorder and clinically significant symptoms (also referred to as perinatal sleep disruption) in the third trimester of pregnancy [3]. Based on data from clinician-administered interviews, the authors found that half of cases with insomnia in late pregnancy are comprised of women who meet diagnostic criteria for the disorder, whereas the other half of women with insomnia experience clinically significant symptoms without meeting diagnostic criteria. Specifically, they estimated that 16-20% of pregnant women meet diagnostic criteria for DSM-5 insomnia disorder in the third trimester, whereas about 17% of pregnant women experience clinically significant insomnia symptoms. As insomnia disorder confers greater morbidity and poorer prognosis than insomnia symptoms [20–22], investigating the disorder in pregnancy will enhance our understanding of the disease burden in peripartum.

Another contributing factor to varying classification methods is that there have been no published validation studies examining the psychometric properties of clinical cut-points on commonly used insomnia symptom measures for detecting insomnia disorder cases in pregnancy. Diagnostic criteria for insomnia disorder, as defined by the Diagnostic Statistical Manual of Mental Disorders 5^th^ Edition (DSM-5) [23], includes complaints of dissatisfaction with difficulty falling asleep, difficulty staying asleep, and/or waking too early. These nocturnal sleep symptoms must occur ≥ 3 nights per week, and cause daytime impairment (e.g., fatigue, impaired work performance). Per the DSM-5, insomnia disorder is specified as *episodic* if its duration is 1-2 months, whereas it is *persistent* when duration is ≥ 3 months. The International Classification of Sleep Disorders, 3^rd^ Edition [24] defines insomnia disorder using similar criteria. Given the diagnostic reliance on self-report symptom severity measures to classify cases of insomnia in pregnancy, there is a critical need to determine the best methods to identify cases using these measures to detect DSM-5 insomnia disorder in this population. Indeed, a lack of standardization of case identification methods has resulted in a wide range of rate estimates between 14% and 62% [1].

Along these lines, nocturnal hyperarousal is a central feature of etiological and maintenance models of insomnia disorder [25–27]. The presleep arousal scale (PSAS) is a commonly used and efficient assessment tool for capturing cognitive and somatic arousal symptoms at night [28]. Although clinical cut-points have been empirically derived for the PSAS in a sample of nonperinatal young adults [29], there have been no validation studies examining clinically significant cut-points for the PSAS in perinatal women. This is a missed opportunity because rapidly growing evidence demonstrates that nocturnal arousal—particularly in the cognitive domain—during peripartum plays a key role in insomnia development and persistence [13], and even in treatment response to insomnia therapy during pregnancy [30,31]. Moreover, nocturnal cognitive arousal influences depression [13], suicidal ideation [16], and mother-to-infant bonding [19] independently of insomnia. To be able to identify and intervene to improve perinatal health, psychometric data are needed to guide accurate case identification of perinatal women with high nocturnal cognitive arousal and high nocturnal somatic arousal.

The present study examined cross-sectional data on insomnia, depression, suicidal ideation, cognitive arousal, and somatic arousal in addition to sociodemographic and other health information from pregnant women. We sought to achieve three goals. *Study Goal #1*: We described rates of DSM-5 insomnia disorder in pregnancy, and compared sociodemographics, sleep-wake symptoms, and mental health factors between women with and without insomnia disorder. *Study Goal #2*: We identified empirically derived cut-points on common validated and standardized insomnia and sleep disturbance measures (Insomnia Severity Index [32] and Pittsburgh Sleep Quality Index [33]) for detecting DSM-5 insomnia disorder cases in pregnant women. Although these measures are commonly used in pregnant samples, investigators have relied on cut-points derived from nonperinatal samples that have not yet been evaluated for clinical validity in pregnancy. Moreover, selection of cutoffs on these measures has varied across studies, resulting in discrepant frequency rate estimates [1]. *Study Goal #3*: We identified cut-points for nocturnal cognitive arousal and nocturnal somatic arousal (via the Presleep Arousal Scale) that correspond to insomnia, depression, and suicidal ideation. After these cut-points were empirically derived, we explored whether cognitive and somatic arousal differentially associated with difficulties of prolonged sleep latency, maintenance, and early morning awakenings.

## Methods

### Study design and sample

This was a cross-sectional analysis of patient-reported data collected as part of a screening process for an in-lab sleep study (sleep study methods and results are not reported here). We emailed an online advertisement for an in-lab study on sleep and health to 1,705 pregnant women receiving care in a multi-hospital health system centrally located in Metro Detroit, Michigan USA. A total of 281 pregnant women contacted our team. After learning study details, 100 women consented to the study and reported patient information via an online survey battery on demographics, sleep, mental health, and other health data. Of these 100 women, 99 provided analyzable data (one participant reported only partial demographic information and no health information before discontinuing). This study was approved by the Internal Review Board at the Henry Ford Health System.

### Measures

Sociodemographic and health information included age, race, annual household income, prior birth history, gestational age, history of prenatal loss, current medication use, body mass index (BMI; derived from electronic medical records), patient-reported diagnoses of any sleep disorders, diabetes, hypertension, and pre-eclampsia.

*Snoring* was assessed via a single binary item (*Do you snore?* Yes/No).

*Insomnia disorder* was assessed per patient-reported symptoms in accordance with criteria from the Diagnostic and Statistical Manual of Mental Disorders, 5^th^ Edition (DSM-5). DSM-5 insomnia disorder requires sleep disturbance ≥ 3 nights/week that results in daytime impairment and that lasts for at least three month for diagnosis. However, as we are interested in cases with insomnia that onset prior to and during pregnancy, we included cases based on a duration cutoff of ≥1 month (which is more aligned with the DSM-IV-TR). Notably, the DSM-5 describes cases with insomnia meeting all criteria but lasting 1-2 months as ‘episodic’ and should be coded as an ‘other specified insomnia disorder.’ To determine sleep disturbance, we asked women if they experienced difficulty falling asleep (sleep latency: >30 minutes to fall asleep), staying asleep (wake after sleep onset: >30 minutes awake in the middle of the night), and waking up too early (early morning awakenings: >30 minutes too early and unable to fall back asleep) for 3 or more nights per week [34–36]. Women who endorsed any of these sleep symptoms ≥3 nights per week met the sleep disturbance criterion. Women were also asked to indicate to what extent sleep problems interfered with their daytime functioning (4-point Likert-type scale: not at all; somewhat; moderately; very much). Those who indicated ‘somewhat,’ ‘moderately,’ or ‘very much’ met the daytime impairment criterion. Patients then reported the duration of their insomnia symptoms as < 1 month, 1-2 months, or ≥3 months. Lastly, patients had to report spending 7 or more hours in bed at night to meet Criterion E (adequate opportunity for sleep). Notably, the DSM-5 does not define adequate opportunity, but ≥7 hours is consistent with the example used in the Duke Structured Clinical Interview for Sleep Disorders – Revised [37]. Patients who endorsed sleep disturbance and daytime impairment criteria and reported a duration of 1 month or longer, despite adequate opportunity for sleep, were classified as having DSM-5 insomnia disorder, which includes patients with ‘episodic’ (1-2 months) or ‘persistent’ (≥ 3 months) courses per DSM-5 specifiers. This technique for detecting DSM-5 insomnia disorder has been supported by large-scale epidemiological studies [38,39].

*Good sleep* was operationalized based on patient responses to DSM-5 questions. Specifically, women who did not meet DSM-5 insomnia disorder criteria, and specifically denied Criterion A (no difficulties falling asleep, staying asleep, or waking up too early) were operationalized as having good sleep.

*Insomnia symptoms* were measured using the Insomnia Severity Index (ISI) [32,40]. Scores range from 0 to 28 with higher scores indicating greater severity. In the original validation study, the ISI ≥ 15 cutoff was identified as indicating clinical insomnia. A psychometric re-evaluation of the ISI in community and clinical samples of nonperinatal adults identified cutoff values of ISI ≥ 10 (community) and ISI ≥ 11 (clinical) as superior cut-points to detect clinical insomnia [40]. The ISI showed high internal consistency in the present sample (Cronbach’s α = .90).

*DSM-5 insomnia disorder per the ISI.* In addition to the traditional quantitative cutoffs, we scored the ISI in accordance with DSM-5 insomnia disorder criteria. This operationalization is referred to as ISI-DSM-5 hereafter. DSM-5 insomnia disorder Criterion A (sleep disturbance) was operationalized as endorsing moderate or worse difficulties falling and/or staying asleep and/or waking up too early (i.e., ISI1, ISI2, and/or ISI3 ≥ 2). Criterion B was operationalized as endorsing sleep-related daytime impairment that is noticeable to others, results in worry/distress, and/or interferes with daily functioning (i.e., ISI5, ISI6, and/or ISI7 ≥ 2). In addition, patients were required to report dissatisfaction with their sleep (i.e., ISI4 ≥ 2). Patients who endorsed Criteria A and B and dissatisfaction with their sleep for a reported duration of ≥ 1 month (duration item appended to ISI) were classified as having DSM-5 insomnia disorder per the ISI.

*Global sleep quality/sleep disturbance* was measured using the Pittsburgh Sleep Quality Index (PSQI) [33]. The PSQI measures a wide range of sleep parameters over the previous month including sleep duration, sleep latency, sleep aid use, and sleep difficulties related to insomnia, breathing difficulties, environmental stimuli, and other factors. A global cutoff of PSQI > 5 is the original cutoff for differentiating good vs poor sleepers. In addition, a cut-point of PSQI > 8 has empirical support for identifying especially poor sleepers within populations marred by elevated sleep disturbance, including patients who are pregnant and those with cancer or traumatic brain injury [4,41,42].

*Sleep onset insomnia* was assessed via PSQI item 5a, which was operationalized as binary variable indicating difficulty falling asleep within 30 minutes on ≥ 3 nights per week over the prior month. Sleep onset insomnia was specifically evaluated due to its close relationship with cognitive arousal and with depression [43–45].

*Short sleep* was assessed via PSQI item 4a, which assesses patient-reported average nightly total sleep time (i.e., sleep duration). Short sleep was a binary variable that was operationalized as sleeping 6 or fewer hours per night over the prior month.

*Depression and suicidal ideation* were measured using the *Edinburgh Postnatal Depression Scale (EPDS)* [46], a 10-item self-report measure of depressive symptoms validated for pregnancy and postpartum. EPDS scores range from 0 to 30, with higher scores indicating greater severity. A cut-point of EPDS ≥ 10 detects both minor and major depression, whereas EPDS ≥ 13 indicates major depression. The EPDS showed high internal consistency in the present sample (Cronbach’s α = .87). EPDS item #10 assessed SI (*thought of harming myself occurred to me*) such that any endorsement was operationalized as a positive screen.

*Nocturnal cognitive arousal* was measured using the Presleep Arousal Scale—Cognitive factor (PSASC) [28]. Specifically, the PSASC measures trait tendency for cognitive arousal while trying to fall asleep at night. Example items from the PSASC are “review or ponder events of the day” and “can’t shut off thoughts.” Scores range from 8 to 40 with higher scores indicating greater nocturnal cognitive arousal. In a nonperinatal sample, analysis of cutoffs demonstrated that PSASC scores ≥ 19 showed good balance between sensitivity and specificity in predicting insomnia, arousal disposition, and anxiety, whereas PSASC ≥ 16 also demonstrated good utility but with the balance shifted toward sensitivity [47]. The PSASC showed high internal consistency in the present sample (Cronbach’s α = .91).

*Nocturnal perinatal-focused rumination* was measured using a single item that we appended to the PSASC. Specifically, participants were asked how intensely they “worried or had stressful thoughts about your pregnancy or new infant” when attempting to fall asleep. Women who scored 1 (not at all) or 2 (slightly) were considered low on perinatal-focused rumination, whereas women who scored 3 (moderately), 4 (a lot), or 5 (extremely) were considered high on perinatal-focused rumination (the median was 2).

*Nocturnal somatic arousal* was measured using the Presleep Arousal Scale—Somatic factor (PSASS) [28]. The PSASS measures trait tendency for somatic arousal while trying to fall asleep at night. Example items from the PSASS are “a jittery, nervous feeling in your body” and “heart racing, pounding, or beating irregularly.” Scores range from 8 to 40 with higher scores indicating greater nocturnal somatic arousal. PSASS cut-points of ≥12 and ≥14 have been empirically supported in the nonperinatal population [29]. The PSASS showed high internal consistency in the present sample (Cronbach’s α = .83).

*Daytime sleepiness* was assessed using the Epworth Sleepiness Scale (ESS) [48], a self-report measure shown to distinguish between individuals with and without sleep disorders in clinical samples [49] and the general population [50]. The ESS showed high internal consistency in the present sample (Cronbach’s α = .84). Higher scores on the ESS indicate more excessive sleepiness, and previous research has used a score of 10 or higher to reflect excessive levels of sleepiness [51,52]. As such, participants in this study with ESS scores ≥10 were classified as having excessive sleepiness.

### Analysis plan

Analyses were conducted using SPSS 25. We first characterized sociodemographics, pregnancy-related information, sleep symptoms, and mental health factors for the full sample. *To achieve Study Goal #1*, we calculated descriptive metrics along with a series of independent samples t-tests to compare women with and without DSM-5 insomnia disorder on sociodemographic factors, and sleep-wake and mental health symptoms.

*To achieve Study Goal #2*, we conducted receiver operating characteristic (ROC) curve analyses to determine appropriate cut-points on the ISI and PSQI for detecting DSM-5 perinatal insomnia disorder. 

For the ROC curve analyses, we reported area under the curve (AUC), sensitivity, specificity, positive predictive value (PPV), and negative predictive value (NPV). AUC values ≥.90 were considered outstanding, ≥.80 were considered excellent, ≥.70 were considered adequate, and <.70 were inadequate per recommendations [53]. For consistency of descriptors, we adopted these thresholds for sensitivity and specificity. When evaluating cutoff performance, we considered Youden’s J statistics (*J = sensitivity + specificity – 1*). Although higher Youden’s J statistic values generally indicate better performance, we also considered specific values of sensitivity and specificity for evaluating cutoff performance, with preference for cut-points that yield acceptable (.70) or better sensitivity and specificity.

We repeated this analytic process for identifying ISI and PSQI cutoffs for detecting good sleep. 

*To achieve Study Goal #3*, we conducted ROC curve analyses for identifying PSASC and PSASS cutoffs indicative of DSM-5 insomnia disorder, sleep onset insomnia, depression, and SI. While clinically relevant cutoffs for the PSASC and PSASS have been identified in one study in the nonperinatal literature [29], these cutoffs do not have the same widespread support as the ISI and PSQI cutoffs. Moreover, the present study consisted of state variables not examined in the previous report. For these reasons, we took a more exploratory approach with regard to PSAS.

Lastly, we conducted exploratory multivariate logistic regression to examine independent associations of cognitive and somatic arousal with the three specific insomnia symptoms: sleep latency, wake after sleep onset, and early morning awakenings as defined by DSM-5 insomnia disorder criteria.

## Results

### Sample characteristics

Participant ages ranged from 18 to 39 years. Although patients from all three trimesters participated in the study, most women were in the second trimester (*n* = 85/99; 85.9%), whereas fewer women were in the first (*n* = 4/99; 4.0%) and third (*n* = 10/99; 10.1%) trimesters. Most participants self-identified racially as non-Hispanic White (*n* = 47/99; 47.5%) or non-Hispanic Black (*n* = 40/99; 40.4%). Antidepressant and sleep aid use at time of assessment was low (*n* = 3/99; 3.0% each). See Table 1 for additional demographic and health-related information.

**Table 1.** Sample demographics and characteristics for all patients and by DSM-5 insomnia disorder classification

	All subjects	Insomnia	No Insomnia	
Sample size	99	19	80	Frequency rate: 19.2%
Age (M ± SD, range)	29.84 ± 4.39, 18-39	27.95 ± 4.71	30.29 ± 4.22	t = –2.13, *p* = .036, Cohen’s d = .52
Poverty (*n*,%)	16/98; 16.3%	5/19; 26.3%	11/79; 13.9%	χ ^2^ = 1.72, *p* = .189
Gestational Week (M ± SD, range)	21.18 ± 4.14, 6-31	20.26 ± 4.15	21.40 ± 4.15	t = –1.08, *p* = .284
BMI (M ± SD)	30.43 ± 8.29	31.23 ± 8.43	30.22 ± 8.30	t = –.47, *p* = .640
Multiparous (*n*;%)	63/99; 63.6%	13; 68.4%	50; 62.5%	χ ^2^ = 0.23, *p* = .630
Prior prenatal loss	25; 25.3%	5; 26.3%	20; 25.0%	χ ^2^ = 0.01, *p* = .906
Antidepressants	3/99; 3.0%	1; 5.3%	2; 2.5%	χ ^2^ = 0.40, *p* = .528
Sleep aids	3/99; 3.0%	0; 0.0%	3; 3.8%	χ ^2^ = 0.74, *p* = .391
*Race (n;%)*				
White	47; 47.5%	7; 36.8%	40; 50.0%	
Black	40; 40.4%	10; 52.6%	30; 37.5%	
Asian	5; 5.1%	0; 0.0%	5; 6.3%	
Middle Eastern or Arabic	1; 1.0%	0; 0.0%	1; 1.3%	
Multiracial	6; 6.1%	2; 10.0%	4; 5.0%	
*Clinical symptoms*				
Snoring (*n*;%)	29/99; 29.3%	5; 26.3%	24; 30.0%	χ ^2^ = 0.10, *p* = .751
ISI (M ± SD)	7.39 ± 5.36	13.89 ± 4.18	5.85 ± 4.37	t = 7.28, *p* < .001, Cohen’s d = 1.88
PSQI (M ± SD)	4.71 ± 2.72	8.00 ± 2.33	3.91 ± 2.15	t = 87.34, *p* < .001, Cohen’s d = 1.82
Sleep Onset Insomnia (*n*;%)	11; 11.1%	8; 42.1%	3; 3.8%	χ ^2^ = 22.87, *p* < .001, RR = 11.08
Sleep latency (mins; M ± SD)	23.23 ± 16.80	34.47 ± 24.77	20.56 ± 13.15	t = 3.42, *p* = .001, Cohen’s d = .70
Sleep duration (hrs; M ± SD)	7.17 ± 1.24	6.26 ± 1.10	7.39 ± 1.17	t = –3.80, *p* < .001, Cohen’s d = 1.00
Short sleep (≤ 6 hrs, *n*;%)	24; 24.2%	13; 68.4%	11; 13.8%	χ ^2^ = 24.99, *p* < .001, RR = 4.96
ESS (M ± SD)	7.34 ± 4.55	11.37 ± 3.59	6.39 ± 4.23	t = 4.74, *p* < .001, Cohen’s d = 1.27
ESS≥10 (*n*;%)	26; 26.35	13; 68.4%	13; 16.3%	χ ^2^ = 21.58, *p* < .001, RR = 4.20
EPDS (M ± SD)	5.45 ± 4.54	9.00 ± 4.15	4.61 ± 4.24	t = 4.07, *p* < .001, Cohen’s d = 1.05
EPDS≥ 10 (*n*;%)	19; 19.2%	10; 52.6%	9; 11.3%	χ ^2^ = 16.95, *p* < .001, RR = 4.65
EPDS≥ 13 (*n*;%)	10; 10.1%	4; 21.1%	6; 7.5%	χ ^2^ = 3.11, *p* = .078, RR = 2.81
Suicidal ideation (*n*;%)	6; 6.1%	4; 21.1%	2; 2.5%	χ ^2^ = 9.28, *p* = .002, RR = 8.44
PSASC (M ± SD)	15.34 ± 6.46	22.74 ± 7.02	13.59 ± 4.93	t = 6.66, *p* < .001, Cohen’s d = 1.51
PFR (M ± SD)	2.21 ± 1.11	2.95 ± 1.13	2.04 ± 1.04	t = 3.38, *p* < .001, Cohen’s d = .84
PSASS (M ± SD)	11.45 ± 4.36	16.26 ± 6.56	10.26 ± 2.53	t = 6.66, *p* < .001, Cohen’s d = 1.21

M ± SD, mean and standard deviation; ISI, insomnia severity index; PSQI, Pittsburgh sleep quality index; mins, minutes; hrs, hours; ESS, Epworth sleepiness scale; EPDS, Edinburgh postnatal depression scale; PSASC, presleep arousal scale, cognitive factor; PFR, perinatal-focused rumination; PSASS, presleep arousal scale, somatic factor; t, t-statistic for independent samples t test; χ ^2^, chi-square; *p*, significance value; RR, risk ratio.

Few women reported sleep disorder diagnoses or cardiometabolic disorders. Just one participant reported an insomnia diagnosis (*n* = 1/99; 1.0%), and five reported a sleep apnea diagnosis (*n* = 5/99; 5.1%). Nine women reported a hypertension diagnosis (*n* = 9/99; 9.1%), just two of these cases were of gestational onset. Three women reported a diabetes diagnosis (*n* = 3/99; 3.0%), and just one of these cases was of gestational onset. Five women reported being diagnosed with pre-eclampsia (*n* = 5/99; 5.1%).

#### DSM-5 insomnia disorder.

We observed a DSM-5 insomnia disorder rate of 19.2% (*n* = 19/99). Among the 19 pregnant women with insomnia disorder, 7 reported acute duration (1–2 months; 7.1% of full sample), and 12 reported chronic duration (≥3 months; 12.1% of full sample). The most commonly endorsed sleep complaint was early morning awakenings (*n* = 14/19; 73.7%), followed by wake after sleep onset (*n* = 12/19; 63.2%) and prolonged sleep latency (*n* = 7/23; 43.5%).

See Table 1 for comparisons of sleep-wake parameters between groups. As expected, patient-reported insomnia symptom severity and global sleep disturbance severity were much higher among women with DSM-5 insomnia disorder relative to those without insomnia. Relative to women without insomnia, women with insomnia disorder reported longer sleep latency (42.1% vs 3.8% reported taking >30 mins to fall asleep ≥ 3 nights/week; *n* = 8/19 vs *n* = 3/80) and shorter nightly sleep duration (by 68 minutes/night) such that they were five times more likely to endorse nightly short sleep (≤ 6 hrs/night). Group comparisons on PSQI component scores revealed large group differences in sleep quality, sleep latency, sleep duration, sleep efficiency, daytime impairment, and disturbances, but no difference in sleep medication use (Supplementary Table S1). Patients with insomnia disorder were four times more likely to screen positive for excessive daytime sleepiness as good sleepers (68.4% vs 16.3%; *n* = 13/19 vs *n* = 13/80).

Regarding mental health, rates of minor/major depression (EPDS ≥ 10) and SI were 4–5 times higher among women with insomnia disorder relative to those without insomnia (see Table 1 for full mental health comparisons). Major depression (EPDS ≥ 13) was three times higher among patients with insomnia. Insomnia was also positively associated with arousal indices. Women with insomnia disorder reported greater nocturnal cognitive arousal, perinatal-focused rumination, and nocturnal somatic arousal relative to women without insomnia; all of which corresponded to large effect sizes (Table 1).

### Detecting DSM-5 insomnia disorder with the ISI and PSQI

Next, we sought to identify clinical cutoffs on the ISI and PSQI for detecting DSM-5 insomnia disorder. Both the ISI (AUC = .913) and PSQI (AUC = .911) demonstrated outstanding accuracy for classifying DSM-5 insomnia disorder. Sensitivity, specificity, PPV, and NPV data are reported in Table 2.

**Table 2.** Validating ISI and PSQI cutoffs to detect DSM-5 insomnia disorder

	Sens	Spec	Youden’s J	PPV	NPV
ISI-DSM-5	.895	.800	.695	17/33; 51.5%	64/66; 97.0%
ISI ≥ 10	.842	.812	.654	16/31; 51.6%	65/68; 95.6%
ISI ≥ 11	.789	.937	.726	15/24; 62.5%	71/75; 94.7%
ISI ≥ 15	.368	.950	.318	10/11; 90.9%	75/88; 85.2%
PSQI > 5	.842	.795	.637	16/32; 50.0%	62/65; 95.4%
PSQI > 8	.500	.684	.184	6/8; 75.0%	76/89; 85.4%

Although we evaluated several ISI and PSQI cut-points for detecting DSM-5 insomnia, we only present data for previously supported cutoffs in this table for two reasons: (1) values not reported here were not identified as strong cut-points for DSM-5 insomnia disorder, therefore (2) we did not include them here to ease readability of the table. Sensitivity: True Positives/Actual Condition Positives. Specificity: True Negatives/Actual Condition Negatives. Youden’s J, Sensitivity + Specificity – 1; PPV, positive predictive value (True Positive/[True Positives + False Positives]); NPV, negative predictive value (True Negatives/True Negatives + False Negatives]); ISI, Insomnia Severity Index; ISI-DSM-5: ISI scored per DSM-5 criteria; PSQI, Pittsburgh Sleep Quality Index; Sens, sensitivity; Spec, specificity.

For the ISI, three ISI cutoffs (ISI-DSM-5, ISI ≥ 10, and ISI ≥ 11) exhibited good clinical utility. Among these three cut-points, the ISI ≥ 11 cut-point yielded the highest Youden’s J index, which reflected outstanding specificity and adequate sensitivity. The ISI-DSM-5 classification yielded the second highest Youden’s J index, which reflected excellent sensitivity and specificity (this cutoff was the most sensitive). The ISI ≥ 10 cut-point yielded the third highest Youden’s J and exhibited both excellent sensitivity and specificity. On the other hand, the original ISI ≥ 15 cutoff for clinical insomnia yielded very poor sensitivity indicating poor clinical utility, despite outstanding specificity.

Regarding the PSQI, the > 5 cutoff yielded excellent sensitivity and adequate specificity, which exhibited a Youden’s J of similar magnitude as the ISI ≥ 10 cutoff. The PSQI > 8, however, produced inadequate sensitivity and specificity. See Supplementary Table S2 for descriptive comparisons of clinical symptoms between women with and without insomnia across each of the four validated cut-points.

#### Posthoc: ISI misclassification of insomnia.

Four of seven items on the ISI do not assess sleep directly. Thus, it is plausible that some positive cases above quantitative cut-points may not actually endorse significant nighttime sleep problems, but still endorse dissatisfaction or distress about their sleep. By design, the ISI-DSM-5 classification method did not positively identify any cases as having insomnia without endorsing nighttime symptoms. However, for the ISI ≥ 10 cutoff, 12.9% of positively identified cases (n=4/31) did not endorse sleep problems on the ISI. For the ISI ≥ 11 cutoff, 4.2% of women did not report sleep problems (n=1/24) on the ISI. See Supplementary Materials for analytic details.

#### Detecting good sleep with the ISI and PSQI.

Next, we evaluated ISI and PSQI cutoffs for detecting good sleep. Both the ISI (AUC = .881) and PSQI (AUC = .893) demonstrated excellent accuracy for classifying good sleepers. Sensitivity, specificity, PPV, and NPV data are reported in Table 3. All four evaluated ISI and PSQI cut-points demonstrated good clinical utility. The ISI-DSM-5 cut-point produced the highest Youden’s J by yielding excellent sensitivity (.873) and specificity (.857).

**Table 3.** Evaluating ISI cutoffs for good sleep (*n* = 71/99) (ISI AUC = .880; PSQI AUC = .893)

	Sens	Spec	Youden’s J	PPV	NPV
ISI-DSM-5	.873	.857	.730	62/66; 93.9%	24/33; 72.7%
ISI ≤ 7	.700	.889	.589	50/53; 94.3%	25/46; 54.3%
ISI ≤ 9	.829	.704	.533	59/68; 86.8%	19/31; 61.3%
PSQI ≤ 5	.843	.778	.621	59/65; 90.8%	21/32; 65.6%

Although we evaluated several ISI and PSQI cut-points for detecting good, we only present data for previously supported cutoffs in this table for two reasons: (1) values not reported here were not identified as strong cut-points for good sleep, therefore (2) we did not include them here to ease readability of the table. Sensitivity: True Positives/Actual Condition Positives. Specificity: True Negatives/Actual Condition Negatives. Youden’s J, Sensitivity + Specificity – 1; PPV, positive predictive value (True Positive/[True Positives + False Positives]); NPV, negative predictive value (True Negatives/True Negatives + False Negatives]); ISI, Insomnia Severity Index; ISI-DSM-5: ISI scored per DSM-5 criteria; PSQI, Pittsburgh Sleep Quality Index; Sens, sensitivity; Spec, specificity.

The ISI ≤ 7 cut-point showed overall good clinical utility, especially regarding its excellent specificity (.889). However, this cutoff yielded barely acceptable sensitivity (.700), misclassifying 30% of good sleepers. Similarly, the ISI ≤ 9 cutoff also showed good clinical utility, especially for its good sensitivity (.829). However, this cut-point yielded only minimally acceptable specificity (.704).

The PSQI ≤ 5 cutoff exhibited excellent sensitivity (.843) and adequate specificity (.778). Notably, this cutoff yielded the second highest Youden’s J of the four methods evaluated for detecting good sleep.

### Identifying clinically relevant cutoffs for nocturnal arousal

Lastly, we empirically derived clinically relevant cutoffs on the Presleep Arousal Scale’s cognitive and somatic arousal factors that correspond to insomnia disorder, sleep onset insomnia, depression, and suicidal ideation during pregnancy.

#### Cognitive arousal.

As an indicator of nocturnal cognitive arousal, the PSASC demonstrated excellent accuracy for identifying insomnia and depression, and outstanding accuracy for detecting SI (Table 4). When examining cut-points, PSASC ≥ 18 performed consistently better than other values. For classifying cases for the five outcomes, PSASC ≥ 18 had the highest Youden’s J for four outcomes (DSM-5 insomnia disorder, sleep onset insomnia, EDPS≥10, and SI), and had the second highest Youden’s J once (EPDS ≥ 13). Importantly, while PSASC ≥ 18 did not produce the highest Youden’s J for major depression, it yielded acceptable or better sensitivity and specificity for major depression, whereas the cut-point with the highest Youden’s J (PSASC ≥ 16) did not (Table 4). Therefore, PSASC ≥ 18 was determined to be the best cut-point. For descriptive purposes, we also compared women with high vs low nocturnal cognitive arousal on sleep-wake and mental health symptoms (Table S3).

**Table 4.** ROC curve analyses for PSASC and PSASS predicting insomnia, depression, and suicidal ideation

	DSM-5 Insomnia			Sleep Onset Insomnia			EPDS ≥ 10			EPDS ≥ 13			Suicidal Ideation		
	AUC = .871			AUC = .866			AUC = .823			AUC = .872			AUC = .900		
*Cognitive*	Sens	Spec	J	Sens	Spec	J	Sens	Spec	J	Sens	Spec	J	Sens	Spec	J
PSASC ≥ 16	.789	.714	.503	.900	.674	.574	.765	.696	.461	1.000	.686	.686	1.000	.656	.656
PSASC ≥ 17	.789	.753	.542	.900	.709	.609	.706	.722	.428	.900	.709	.609	1.000	.689	.689
PSASC ≥ 18	.789	.792	.581	.900	.744	.644	.706	.759	.465	.900	.744	.644	1.000	.722	.722
PSASC ≥ 19	.737	.844	.581	.700	.779	.479	.647	.810	.457	.800	.791	.591	.833	.767	.600
	AUC = .829			AUC = .838			AUC = .819			AUC = .812			AUC = .728		
*Somatic*	Sens	Spec	J	Sens	Spec	J	Sens	Spec	J	Sens	Spec	J	Sens	Spec	J
PSASS ≥ 11	.842	.649	.491	.900	.605	.505	.824	.633	.457	.800	.593	.393	.667	.567	.234
PSASS ≥ 12	.789	.779	.568	.800	.621	.421	.765	.759	.524	.800	.721	.521	.667	.689	.356
PSASS ≥ 13	.737	.831	.568	.700	.767	.467	.765	.823	.588	.800	.779	.579	.667	.744	.411
PSASS ≥ 14	.579	.922	.501	.700	.826	.562	.647	.861	.508	.700	.826	.526	.667	.800	.467

DSM-5 insomnia disorder = Insomnia disorder of ≥ 1 month per the Diagnostic and Statistical Manual of Mental Disorders, 5

^th^ Edition. Sleep onset insomnia assessed via the Pittsburgh Sleep Quality Index item #5_1, operationalized as having difficulty falling asleep within 30 minutes for ≥ 3 nights/week over the prior month. EPDS = Edinburgh Postnatal Depression Scale; EPDS ≥ 10 indicates minor or major depression. EPDS ≥ 13 indicates major depression. Suicidal Ideation assessed via the EPDS item #10; responses dichotomized such that any endorsement is considered a positive screen for suicidal ideation. PSASC, Presleep Arousal Scale, Cognitive Factor; PSASS, Presleep Arousal Scale, Somatic Factor; AUC, area under the curve; Sens, Sensitivity; Spec, Specificity; For AUC, Sens, and Spec, we used the following thresholds: ≥.900 = outstanding; ≥.800 = excellent. ≥.700 = acceptable; <.700 = unacceptable; J, Youden’s J index (Sens+Spec-1).

#### Somatic arousal.

The PSASS demonstrated excellent accuracy for predicting insomnia and depression, but only adequate accuracy for predicting SI (Table 4). A cutoff of PSASS ≥ 13 yielded the highest Youden’s J indices for three outcomes: DSM-5 insomnia, EPDS ≥ 10, and EPDS ≥ 13. Although it did not produce the highest Youden’s J for sleep onset insomnia (PSASS ≥ 14 performed best here), sensitivity and specificity for PSASC ≥13 were adequate. Regarding SI, no PSASS cut-point yielded both acceptable (or better) sensitivity and specificity. For descriptive purposes, we compared women with high vs low somatic arousal on sleep-wake and mental health symptoms (Supplementary Table S3).

### Specificity of nighttime sleep symptoms with nocturnal cognitive and somatic arousal

Lastly, we explored whether nighttime insomnia symptoms (sleep latency, wake after sleep onset, early morning awakenings) differentially associated with cognitive and somatic arousal. We performed three multivariate logistic regression models wherein sleep latency, sleep maintenance, and early morning awakening symptoms (binary variables in accordance with DSM-5 insomnia disorder Criterion A) were regressed on PSASC ≥ 18 and PSASS ≥ 13 (See Table 5 for full results). Analyses revealed that high cognitive arousal, but not somatic arousal, was independently associated with increased odds for sleep latency symptoms (OR = 4.51) and wake after sleep onset symptoms (OR = 5.65). In contrast, high somatic arousal, but not cognitive arousal, was significantly and independently associated with early morning awakenings (OR = 10.33).

**Table 5.** Multivariate logistic regression models regressing sleep latency, wake after sleep onset, and early morning awakenings on high cognitive and somatic arousal

	OR	95% CI	*p*
***SL***			
PSASC ≥ 18	4.51	1.03, 19.76	.046
PSASS ≥ 13	1.39	0.33, 5.91	.654
***WASO***			
PSASC ≥ 18	5.65	1.39, 22.99	.016
PSASS ≥ 13	2.29	0.60, 8.76	.225
***EMA***			
PSASC ≥ 18	1.80	0.49, 6.66	.377
PSASS ≥ 13	10.33	2.74, 38.90	.001

SL, sleep latency; WASO, wake after sleep onset; EMA, early morning awakening; PSASC, presleep arousal scale cognitive factor; PSASS, presleep arousal scale somatic factor; OR, Odds ratio; 95% CI, 95% confidence interval for the OR; *p*, significance value.

## Discussion

### DSM-5 insomnia disorder in pregnancy

In a sample of 99 women predominantly in mid-pregnancy, 19 women met diagnostic criteria for DSM-5 insomnia disorder (19.2%). As classification methods derived from severity-based symptom surveys can overestimate prevalence, it is likely that higher prevalence rates reported in other studies capture both insomnia disorder and clinically significant insomnia symptoms. Indeed, our estimated rate of 19.2% was lower than a recent meta-analytic estimate of 27.2% prevalence of clinically significant insomnia in the second trimester [1]. However, our rate of 19.2% is highly consistent with a recent estimate of 16-20% for DSM-5 insomnia disorder in late pregnancy as diagnosed by clinical interview [3]. These data suggest that our operationalization of survey-assessed DSM-5 insomnia disorder yields estimates consistent with rates derived from clinical interviews; this finding may inform epidemiological efforts in this patient population going forward.

Importantly, DSM-5 insomnia disorder rates reported in the present study and in a previous study utilizing clinician-administered interviews [3] suggests that insomnia disorder rates are twice as high as epidemiological estimates of DSM- and ICSD-based insomnia disorder rates in the broader population [20,21], thereby highlighting the disproportionate burden of insomnia disorder for pregnant women.

For the present study, it is worth highlighting that we required symptom duration of just one month to be classified as having DSM-5 insomnia disorder. In the DSM-5, an ‘insomnia disorder’ diagnosis requires three months or longer duration, whereas insomnia lasting 1-2 months is considered ‘episodic’ and as an ‘other specified insomnia disorder.’ Importantly, we included cases with one month or longer duration because requiring three months duration would rule out many women with gestational onset of insomnia, thereby under-estimating insomnia rates in pregnancy. Notably, the observed rate of DSM-5 insomnia disorder when requiring three months duration in the present study was 12.1%, which is similar to prevalence rates in the general population. Future research should investigate whether timing of insomnia disorder onset (gestational vs prepregnancy) influences morbidity trajectories.

#### Comorbid symptoms.

Women with insomnia disorder, relative to those without, reported 5-8 times greater likelihood of reporting short sleep (≤ 6 hrs/night), depression, and suicidal thoughts. Further, pregnant women with insomnia disorder reported greater cognitive arousal, somatic arousal, and perinatal-focused rumination than women without insomnia; all large effects. The pattern of these findings is consistent with prior investigations in pregnancy that classified insomnia based on symptom severity rather than DSM-5 criteria [4,8,13,45,54–61], which supports both insomnia classification methods (diagnostic criteria and severity-based) for capturing the prenatal insomnia experience including morbidity and comorbidities.

### ISI cutoffs to detect DSM-5 insomnia disorder

Sensitivity and specificity metrics supported three methods for classifying cases with insomnia. The ISI ≥ 11 cut-point yielded the highest Youden’s J as reflected by outstanding specificity and adequate sensitivity. By comparison, our scoring method for the ISI we designed to align with DSM-5 diagnostic criteria (see *DSM-5 insomnia disorder per the ISI* in the Methods) produced the second highest Youden’s J as reflected by both excellent sensitivity and specificity. The ISI ≥ 10 produced the third highest Youden’s J, and its sensitivity and specificity were both excellent as well. Important to highlight here is that the two quantitative cutoffs supported here have also been supported in the general population (≥ 10 in community samples, ≥ 11 in clinic samples) [40].

As these three classification methods yielded strong psychometrics, a blanket recommendation of one over the others is inappropriate. Our ISI-DSM-5 method and the ISI ≥ 10 cut-point should be considered when prioritizing sensitivity (e.g., identifying insomniacs who may be at risk for SI), whereas ISI ≥ 11 should be considered when prioritizing specificity. It is worth highlighting that the ISI-DSM-5 scoring method is designed to ensure that positive cases endorse nighttime sleep problems. By comparison, 12.9% of women who scored ISI ≥ 10 did not endorse nighttime sleep problems, whereas 4.2% of women who scored ISI ≥ 11 did not endorse sleep problems.

We want to emphasize that the ISI ≥ 15 cut-point—the original cutoff for clinical insomnia in the broader patient population—yielded very poor sensitivity (.368), resulting in missing half of the cases with insomnia. Furthermore, improvement in specificity from ISI ≥ 11 (.937) to ISI ≥ 15 (.950) was negligible, which means that choosing the 15-point cutoff over the 11-point cutoff substantially sacrifices sensitivity without a meaningful gain in specificity. We strongly advise against using the ISI ≥ 15 cut-point to classify cases with insomnia in pregnancy.

### PSQI cutoffs to detect DSM-5 insomnia disorder

Despite not being an insomnia-specific measure, the PSQI > 5 cutoff yielded excellent sensitivity and adequate specificity for detecting DSM-5 insomnia disorder. On the other hand, the PSQI > 8 cutoff performed very poorly and is not recommended for identifying cases with insomnia in this population. While we recommend the ISI over the PSQI for assessing insomnia in this population, it is notable that the PSQI > 5 cut-point showed very good clinical utility in detecting cases with insomnia and can be used to assess broader sleep complaints and disturbances in this population.

### ISI and PSQI cutoffs to detect good sleep

The detection of good sleep may inform operationalization of insomnia remission in clinical trials. Our custom ISI-DSM-5 classification method yielded both excellent sensitivity and specificity, which resulted in the highest Youden’s J by a considerable amount. These data offer strong support for its detection of good sleep, which may be a valid and balanced indicator of insomnia remission in clinical trials in this patient population.

The ISI ≤ 7 and ISI ≤ 9 cut-points exhibited good psychometrics, but with inverse trade-offs. The ISI ≤ 7 is the most common indicator of remission in insomnia clinical trials. This cut-point yielded excellent specificity, but only barely acceptable sensitivity. Indeed, this classification method missed 30% of good sleepers. In clinical trials, it is possible that this cutoff may underestimate insomnia remission. By contrast, the ISI ≤ 9 cut-point yielded excellent sensitivity, but only acceptable specificity. Although the ISI ≤ 7 yielded a slightly larger Youden’s J, the sensitivity and specificity metrics are so similar (but inverse) between these cutoffs that we do not recommend one over the other. Long-term follow-up data—including patient satisfaction and long-term sleep and mental health symptoms—from clinical trials in this population may elucidate which cutoff is a better indicator of insomnia remission in this population.

Regarding the PSQI, it yielded excellent sensitivity and adequate specificity. Although we do not recommend using the PSQI as a primary indicator of insomnia, the PSQI ≤ 5 cut-point is a valid indicator of good sleep and, by extension, serves as a good indicator of remission from sleep disturbance in a pregnant population.

### Classifying high nocturnal cognitive arousal with the PSASC

The PSASC ≥ 18 cutoff best corresponded to clinically significant sleep-wake and mental health symptoms. Importantly, it was the only PSASC cutoff that yielded adequate or better sensitivity and specificity for each outcome measure. Therefore, we recommend this cut-point for use in this population.

In a nonperinatal sample, Puzino and colleagues recommended a PSASC cutoff of ≥ 16 as the best indicator for arousability, whereas PSASC ≥ 19 best detected sleep onset insomnia, insomnia symptoms, and anxiety [29]. Both of these cut-points performed well in our pregnant sample and should be considered for use in this population when needed to prioritize sensitivity (≥ 16) or specificity (≥ 19).

### Classifying high nocturnal somatic arousal with the PSASS

The PSASS ≥ 13 was the best cutoff for insomnia and depression, exhibiting the highest Youden’s J across outcomes (except suicidal ideation, which we did not consider since no cut-point yielded adequate sensitivity). In the aforementioned Puzino et al study [29], PSASS ≥ 12 best corresponded to insomnia and arousability, whereas PSASS ≥ 14 best corresponded to anxiety. In the present study, the PSASS ≥ 12 and PSASS ≥ 14 cutoffs yielded good psychometrics across most domains. Although we recommend PSASS ≥ 13 for use in this population, the 12- and 14-point cutoffs may be considered when prioritizing sensitivity or specificity.

### Cognitive and somatic arousal correspond to different nocturnal sleep symptoms

Difficulty falling asleep and staying asleep in the middle of the night were uniquely associated with high cognitive arousal, but not with somatic arousal. These findings are consistent with polysomnography and actigraphy data showing that individuals with high nocturnal cognitive arousal take about 45 minutes longer to fall asleep, and are awake for 44 minutes longer in the night relative to those with low cognitive arousal [62,63]. In contrast, high nocturnal somatic arousal was uniquely associated with early morning awakenings, but cognitive arousal was not. These data offer preliminary support that etiological processes for nocturnal insomnia symptoms in pregnancy may differ such that cognitive processes drive early and middle of the night sleep disruptions, whereas somatic processes may underlie terminal insomnia. Future research is needed to further explore these symptom-pattern associations. Further support may guide tailoring of insomnia intervention based on presenting symptom patterns during pregnancy.

### Excessive sleepiness in prenatal insomnia?

An incidental finding was that two out of every three women with DSM-5 insomnia disorder reported excessive daytime sleepiness. For context, population studies estimate that excessive daytime sleepiness (per patient report) in the general population is 20–25% [64]. Thus, our rates that are 3 times higher than the general population emphasize the alarmingly great burden of excessive daytime sleepiness in pregnant women with insomnia. The potential role of sleepiness in prenatal insomnia has many considerations outside the main foci of the present study, thus we discuss this finding in further detail in the Supplementary Materials.

If sleepiness is a daytime impairment of insomnia in pregnancy, then this has profound implications for prenatal insomnia intervention. Cognitive behavioral therapy for insomnia (CBTI) is the only empirically supported treatment for insomnia during pregnancy [65–69]. One component of CBTI is sleep restriction, which reduces time spent in bed to consolidate sleep. This reduction of time-in-bed leads to small short-lived increases in daytime sleepiness, which poses a potential safety risk for patients with CBTI [70]. By extension, safety risk may be further increased in patients who are already excessively sleepy. Thus, safety precautions for this patient population should be considered using an informed approach. Notably, most CBTI trials in pregnant women modified behavioral sleep strategies to protect against sleep deprivation and daytime sleepiness via modifying sleep restriction guidelines and/or allowing strategic naps [65,67–69].

### Limitations

Study findings should be interpreted in light of certain methodological limitations. The first is that DSM-5 insomnia disorder diagnoses were assessed via online survey rather than clinical interview. Clinical interviews are gold standard for diagnosing sleep disorders. Importantly, clinicians are better able to ascertain whether adequate sleep opportunity is sleep conducive and to rule out other potential sleep disorders that may better account for patient complaints of insomnia. Indeed, rates of other sleep disorders, such as obstructive sleep apnea and restless legs syndrome, are elevated in pregnancy and increase as pregnancy progresses [71–74]. As such, it is possible that clinical inquiry may have resulted in different classifications for some patients. As such, future research should determine whether these results replicate when DSM-5 insomnia disorder is diagnosed via clinical interviews. Even so, it is worth emphasizing that our estimated rates of DSM-5 insomnia disorder are consistent with rates derived from clinician-administered diagnostic interviews of pregnant women of similar gestational age [3]. And notably, the technique employed in the present study has been supported by large-scale epidemiological studies [38,39], and the empirically derived quantitative cutoffs identified in this report are consistent with prior psychometric validation of the ISI and PSQI [33,40].

Another important limitation is sampling bias. Participants in this study were interested in enrolling in a multi-night in-lab sleep study. As a result, women with prenatal complications and high-risk pregnancy may have been less likely to be interested in participating in a study that involved sleeping in a lab across multiple nights. If we oversampled healthy women, then it is possible that rates of sleep-wake and mental health conditions may actually be under-estimated.

A third limitation pertains to sample representativeness. While we observed good representation among non-Hispanic white and black women, women of other racial and ethnic backgrounds were underrepresented in this study. Along these lines, pregnant women in the second trimester comprised most of the sample. Although posthoc analyses show that the empirically derived ISI cutoffs perform well in women in the first and third trimesters, these cutoffs should be evaluated in a sample with greater representation in these trimesters. Lastly, we assessed SI via item #10 on the EPDS. While this operationalization is common practice in perinatal samples [16,45,75,76], a more nuanced assessment of suicidality—including ideation severity, ideation intensity, behavior, and lethality (e.g., via the Columbia Suicide Severity Rating Scale [77])—is needed to better understand suicidality in peripartum.

## Conclusion

DSM-5 insomnia disorder affects a large segment of the pregnant population and was associated with elevated depression, suicidality, cognitive and somatic arousal, perinatal-focused rumination, and excessive daytime sleepiness. Although prenatal insomnia has historically gone underrecognized, even for women receiving routine prenatal care [4,12], methods for expedient and accurate case identification are now available and can enhance assessment efforts. When evaluating ISI-based classifications, previously identified cutoffs of ISI ≥ 10 and ISI ≥ 11 showed excellent clinical utility, whereas ISI ≥ 15 did not. A word of caution, however, when using quantitative cutoffs is that some positively identified cases do not endorse significant nighttime symptoms. To circumvent this potential issue, our custom scoring technique ensures that positive cases endorse nighttime sleep problems, and yielded excellent clinical utility for detecting survey-assessed DSM-5 insomnia disorder. Although the PSQI is not an insomnia-specific measure, its 5-point cutoff yielded good utility for detecting insomnia disorder and good sleep. Empirical cut-points identified in this report for clinically significant nocturnal cognitive and somatic arousal may standardize operationalization of high nocturnal arousal in these domains going forward adding to future in perinatal research approaches to insomnia and other conditions.

## Supplementary Material

zpac006_suppl_Supplementary_Material
