# Supplementary material for: DSM-5 insomnia disorder in pregnancy: associations with depression, suicidal ideation, and cognitive and somatic arousal, and identifying clinical cutoffs for detection
Source: Sleep Adv. 2022 Mar 11;3(1):zpac006. doi: 10.1093/sleepadvances/zpac006 (PMC8981986; doi:10.1093/sleepadvances/zpac006)
Supplement: zpac006_suppl_Supplementary_Material [file zpac006_suppl_supplementary_material.docx]

SUPPLEMENTARY MATERIALS: DSM-5 Insomnia Disorder in Pregnancy: Associations with Depression, Suicidal Ideation, and Cognitive and Somatic Arousal, and Identifying Clinical Cutoffs for Detection

*Detecting DSM-5 insomnia disorder with the ISI and PSQI*.

Posthoc: ISI misclassification of insomnia cases. Four of seven items on the ISI do not pertain to nighttime symptoms. Thus, it is possible that some respondents with elevated scores may not endorse nighttime symptoms, thereby precluding insomnia diagnosis. To explore the frequency of this misclassification, we ran frequency rates for nighttime sleep problems (per ISI items 1, 2, and 3 separately, operationalized as ‘moderate’ or worse) among women who endorsed ISI ≥ 10 and ISI ≥ 11. Data showed that, among women who reported ISI scores 10 and above, 87.1% (27/31) reported nighttime sleep symptoms on the ISI (i.e., 12.9% [n=4/31] misclassified). For those who scored 11 and higher, 95.8% (n=23/24) reported nighttime sleep symptoms on the ISI (i.e., 4.2% [n=1/24] misclassified).

Table S1. Pittsburgh Sleep Quality Index differences between participants with and without DSM-5 insomnia disorder.

|  | **Insomnia** | **No Insomnia** | **test, significance** | **Cohen’s d** |
| --- | --- | --- | --- | --- |
| Component 1: Sleep Quality (M±SD) | 1.68±.58 | .93±.59 | t=5.05, p<.001 | 1.28 |
| Component 2: Sleep Latency (M±SD) | 2.00±.88 | 1.03±.80 | t=4.71, p<.001 | 1.15 |
| Component 3: Sleep Duration (M±SD) | 1.11±.66 | .50±.73 | t=3.31, p=.001 | 0.88 |
| Component 4: Sleep Efficiency (M±SD) | 1.32±1.20 | .17±.49 | t=6.53, p<.001 | 1.25 |
| Component 5: Step Disturbances (M±SD) | 1.79±.42 | 1.29±.51 | t=3.99, p<.001 | 1.07 |
| Component 6: Sleep Medication (M±SD) | .00±00 | .09±.43 | t=0.89, p=.376 | -- |
| Component 7: Daytime Dysfunction (M±SD) | 1.79±.71 | .78±.75 | t=5.37, p<.001 | 1.38 |

Insomnia = DSM-5 insomnia disorder. M=mean. SD=standard deviation.

Table S2. Comparing sleep-wake and mental health symptoms per empirically derived ISI and PSQI cutoffs.

|  | **ISI ≥ 10** | **ISI < 10** |  | **ISI ≥ 11** | **ISI < 11** |  |
| --- | --- | --- | --- | --- | --- | --- |
| Sample size | 31 | 68 | Prevalence: 31.3% | 23 | 76 | Prevalence: 23.2% |
| PSQI (M±SD) | 7.03±2.66 | 3.62±1.96 | t=7.10***, d=1.46 | 7.71±2.61 | 3.73±1.92 | t=8.04***, d=1.74 |
| SL (mins; M±SD) | 31.29±24.60 | 19.56±9.92 | t=3.39**, d=.63 | 35.83±26.16 | 19.20±9.66 | t=4.64***, d=.84 |
| TST (hrs; M±SD) | 6.68±1.39 | 7.40±1.09 | t=-2.78*, d=.58 | 6.38±1.20 | 7.43±1.14 | t=-3.88***, d=.90 |
| Short sleep (n;%) | 14; 45.2% | 10; 14.7% | χ^2^=10.75**, RR=3.07 | 13; 54.2% | 11; 14.7% | χ^2^=15.45***, RR=3.69 |
| ESS (M±SD) | 11.10±5.00 | 5.63±3.10 | t=6.66***, d=1.31 | 11.29±4.74 | 6.08±3.71 | t=5.59***, d=1.22 |
| ESS≥10 (n;%) | 18; 58.1% | 8; 11.8% | χ^2^=23.57***, RR=4.92 | 16; 66.7% | 10; 13.3% | χ^2^=23.49***, RR=5.02 |
| EPDS (M±SD) | 8.94±4.00 | 3.87±3.86 | t=6.00***, d=1.29 | 9.67±3.93 | 4.11±3.86 | t=6.11***, d=1.43 |
| EPDS ≥ 10 (n;%) | 15; 48.4% | 4; 5.9% | χ^2^=24.81***, RR=8.20 | 13; 54.2% | 6; 8.0% | χ^2^=24.99***, RR=6.78 |
| EPDS ≥ 13 (n;%) | 7; 22.6% | 3; 4.4% | χ^2^=7.74**, RR=5.14 | 7; 29.2% | 3; 4.0% | χ^2^=12.68***, RR=7.30 |
| SI (n;%) | 5; 16.1% | 1; 1.5% | χ^2^=8.04**, RR=10.73 | 4; 17.4% | 2; 2.6% | χ^2^=6.76**, RR=6.69 |
| PSASC (M±SD) | 20.31±7.28 | 12.94±4.32 | t=6.54***, d=1.23 | 22.29±7.21 | 13.12±4.30 | t=7.61*** d=1.54 |
| PFR (M±SD) | 2.84±1.21 | 1.93±.94 | t=4.09***, d=.84 | 3.17±1.17 | 1.91±.90 | t=5.53***, d=1.21 |
| PSASS (M±SD) | 15.41±5.56 | 9.73±2.09 | t=7.29***, d=1.35 | 16.30±5.80 | 9.92±2.22 | t=7.82***, d=1.45 |
|  |  |  |  |  |  |  |
|  | **ISI-DSM-5 ID** | **No Insomnia** |  | **PSQI > 5** | **PSQI ≤ 5** |  |
| Sample size | 33 | 66 | Prevalence: 33.3% | 32 | 65 | 33.0% |
| PSQI (M±SD) | 7.09±2.41 | 3.54±2.00 | t=7.68***, d=1.60 | -- | -- | -- |
| SL (mins; M±SD) | 33.18±23.38 | 18.26±9.01 | t=4.57*** d=.84 | 34.38±23.03 | 17.54±8.84 | t=5.19***, d=.97 |
| TST (hrs; M±SD) | 6.26±1.10 | 7.63±1.03 | t=-6.09*** d=1.29 | 6.19±1.02 | 7.67±1.02 | t=-6.72***, d=1.45 |
| Short sleep (n;%) | 19; 52.8% | 5; 7.9% | χ^2^=25.08***, RR=6.68 | 18; 56.3% | 5; 7.7% | χ^2^=27.95***, RR=7.32 |
| ESS (M±SD) | 9.88±4.97 | 6.08±3.76 | t=4.25***, d=.86 | 9.81±4.75 | 6.20±4.04 | t=3.92***, d=.82 |
| ESS≥10 (n;%) | 17; 51.5% | 9; 13.6% | χ^2^=16.30***, RR=3.79 | 17; 53.1% | 9; 13.8% | χ^2^=16.86***, RR=3.85 |
| EPDS (M±SD) | 8.09±4.51 | 4.14±3.98 | t=4.46*** d=.93 | 8.22±3.95 | 4.18±4.27 | t=4.48***, d=.98 |
| EPDS ≥ 10 (n;%) | 14; 42.4% | 5; 7.6% | χ^2^=17.23***, RR=5.58 | 12; 37.5% | 7; 10.8% | χ^2^=9.73**, RR=3.47 |
| EPDS ≥ 13 (n;%) | 7; 21.2% | 3; 4.5% | χ^2^=6.73**, RR=4.71 | 5; 15.6% | 5; 7.7% | χ^2^=1.46, p=.227 |
| SI (n;%) | 5; 15.2% | 1; 1.5% | χ^2^=7.19**, RR=10.13 | 4; 12.5% | 2; 3.1% | χ^2^=3.28, p=.070 |
| PSASC (M±SD) | 19.70±7.61 | 13.17±4.47 | t=5.37***, d=1.05 | 20.00±7.33 | 13.14±4.67 | t=5.60***, Cohen’s d=1.12 |
| PFR (M±SD) | 2.91±1.23 | 1.86±.86 | t=4.92***, d=.99 | 2.78±.98 | 1.97±1.08 | t=3.60**, d=.79 |
| PSASS (M±SD) | 14.66±5.69 | 9.84±2.23 | t=5.94***, d=1.12 | 14.16±5.63 | 10.16±2.88 | t=4.57***, d=.89 |

Note: M±SD = mean and standard deviation. PSQI = Pittsburgh sleep quality index. SL = sleep latency. TST = total sleep time (i.e., sleep duration). Short sleep = 6 ≤ hrs/night. mins = minutes. hrs = hours. ESS = Epworth sleepiness scale. EPDS = Edinburgh postnatal depression scale. SI = suicidal ideation. PSASC = pre-sleep arousal scale, cognitive factor. PFR = perinatal-focused rumination. PSASS = pre-sleep arousal scale, somatic factor. t = t-statistic for independent samples t test. χ^2^ = chi-square. p = significance value (reported for nonsignificant findings). **p<.01. ***p<.001. d = Cohen’s d effect size. RR = risk ratio.

*Posthoc Descriptives: Insomnia, depression, and SI in pregnant women with high vs low arousal*

Cognitive arousal. To provide descriptive differences in sleep and mood symptoms based on high and low cognitive arousal, we compared women per our identified PSASC ≥ 18 cutoff. In addition, we repeated these descriptive analyses based on PSASC ≥ 16 (prioritizing sensitivity) and PSASC ≥ 19 (prioritizing specificity), which were identified as a good alternate cut-points in the ROC curve analyses. All three cut-points demonstrated good clinical utility for discriminating between those with and without sleep and mood symptoms (see Table S1 below for results). Overall, the PSASC ≥ 18 cutoff yielded the most significant group differences and the largest effect sizes in group differences.

Somatic arousal. To provide descriptive differences in sleep and mood symptoms based on high and low somatic arousal, we compared women per our identified PSASS ≥ 13 cutoff. In addition, we repeated these descriptive analyses based on PSASS ≥ 12 (prioritizing sensitivity) and PSASS ≥ 14 (prioritizing specificity), which were identified as a good alternate cut-points in the ROC curve analyses. All three cut-points demonstrated good clinical utility (see Table S1 below for results). Overall, both the PSASS ≥ 12 and PSASS ≥ 13 cutoffs performed better than the PSASS ≥ 14 cutoff in regard to yielding the most significant group differences and large effect sizes. However, the PSASS ≥ 13 cutoff performed slightly better than the PSASC ≥ 12 cutoff in regards to effect sizes, and that ≥ 13 identified women at risk for SI whereas the ≥ 12 did not.

Table S2. Descriptive comparisons of pregnant women with high vs low cognitive arousal on sleep and mood symptoms.

|  | **PSASC ≥ 16** | **PSASC < 16** |  | **PSASC ≥ 18** | **PSASC < 18** |  | **PSASC ≥ 19** | **PSASC < 19** |  |
| --- | --- | --- | --- | --- | --- | --- | --- | --- | --- |
| n | 38 | 61 |  | 32 | 65 |  | 27 | 72 |  |
| Poverty | 9/37; 24.3% | 7; 11.5% | χ^2^=2.78, p=.095 | 9/31; 29.0% | 7; 10.4% | χ^2^=5.36*, RR=2.79 | 6/26; 23.1% | 10; 13.9% | χ^2^=1.18, p=.277 |
| Snoring (n;%) | 9; 23.7% | 20; 32.8% | χ^2^=0.94, p=.333 | 8; 25.0% | 21; 31.3% | χ^2^=0.42, p=.517 | 6; 22.2% | 23; 31.9% | χ^2^=0.90, p=.344 |
| DSM-5 Insomnia | 15; 39.5% | 4; 6.6% | χ^2^=16.36***, RR=5.98 | 15; 46.9% | 4; 6.0% | χ^2^=23.37***, RR=7.82 | 14; 51.9% | 5; 6.9% | χ^2^=25.53***, RR=7.52 |
| ISI (M±SD) | 10.61±5.47 | 5.39±4.23 | t=5.32***, d=1.07 | 11.63±5.27 | 5.37±4.10 | t=6.46***, d=1.36 | 11.74±5.57 | 5.76±4.29 | t=5.68***, d=1.20 |
| PSQI (M±SD) | 6.34±2.80 | 3.66±2.08 | t=5.40***, d=1.09 | 6.69±2.88 | 3.74±2.03 | t=5.83***, d=1.18 | 6.78±2.76 | 3.91±2.25 | t=5.26***, d=1.14 |
| SL (M±SD) | 28.95±19.49 | 19.67±13.90 | t=2.76**, d=.55 | 30.78±20.48 | 19.63±13.47 | t=3.24**, d=.64 | 31.85±21.85 | 20.00±13.27 | t=3.28**, d=.66 |
| TST (M±SD) | 6.87±1.21 | 7.36±1.23 | t=-1.96, p=.053 | 6.83±1.27 | 7.34±1.19 | t=-1.94, p=.055 | 6.78±1.32 | 7.32±1.18 | t=-1.97, p=.052 |
| TST ≤ 6 (n;%) | 13; 34.2% | 11; 18.0% | χ^2^=3.34, p=.068 | 12; 37.5% | 12; 17.9% | χ^2^=4.53*, RR=2.09 | 10; 37.0% | 14; 19.4% | χ^2^=3.31, p=069 |
| ESS (M±SD) | 9.63±4.48 | 5.92±4.01 | t=4.29*** d=.87 | 10.13±4.56 | 6.01±3.93 | t=4.62***, d=.97 | 10.30±4.51 | 6.24±4.07 | t=4.29***, d=.95 |
| EPDS (M±SD) | 8.50±4.53 | 3.55±3.39 | t=6.19***, d=1.24 | 8.94±4.49 | 3.79±3.54 | t=6.20***, d=1.27 | 9.19±4.42 | 4.06±3.75 | t=5.77***, d=1.25 |
| EPDS ≥ 10 (n;%) | 14; 36.8% | 5; 8.2% | χ^2^=12.39***, RR=4.49 | 13; 40.6% | 6; 9.0% | χ^2^=14.00***, RR=4.51 | 12; 44.4% | 7; 9.7% | χ^2^=15.27***, RR=4.58 |
| EPDS ≥ 13 (n;%) | 10; 26.3% | 0; 0.0% | χ^2^=17.86*** | 9; 28.1% | 1; 1.5% | χ^2^=16.92***, RR=18.73 | 8; 29.6% | 2; 2.8% | χ^2^=15.59***, RR=10.57 |
| SI (n;%) | 6; 15.8% | 0; 0.0% | χ^2^=10.25*** | 6; 18.8% | 0; 0.0% | χ^2^=13.73*** | 5; 18.5% | 1; 1.4% | χ^2^=10.12***, RR=13.21 |
| PFR (M±SD) | 2.84±1.13 | 1.82±.90 | t=4.97***, d=1.00 | 2.97±1.15 | 1.85±.89 | t=5.30***, d=1.09 | 3.11±1.12 | 1.88±.89 | t=5.67***, d=1.22 |
| PSASS (M±SD) | 14.00±5.62 | 9.85±2.20 | t=5.10***, d=.97 | 14.71±5.80 | 9.89±2.21 | t=5.88***, d=1.10 | 15.00±6.17 | 10.13±2.43 | t=5.58***, d=1.04 |
|  |  |  |  |  |  |  |  |  |  |
|  | **PSASC ≥ 12** | **PSASC < 12** |  | **PSASC ≥ 13** | **PSASC < 13** |  | **PSASC ≥ 14** | **PSASC < 14** |  |
| n | 32 | 64 |  | 27 | 69 |  | 22 | 74 |  |
| Poverty | 10/31; 32.3% | 6; 9.4% | χ^2^=7.81*, RR=3.44 | 8/26; 30.8% | 8; 11.6% | χ^2^=4.96*, RR=2.66 | 5/22; 22.7% | 11; 15.1% | χ^2^=0.71, p=.400 |
| Snoring (n;%) | 13; 40.6% | 15; 23.4% | χ^2^=3.05, p=.081 | 12; 44.4% | 16; 23.2% | χ^2^=4.24*, RR=1.91 | 8; 25.0% | 21; 31.3% | χ^2^=0.42, p=.517 |
| DSM-5 Insomnia | 15; 46.9% | 4; 6.3% | χ^2^=22.18***, RR=7.44 | 14; 51.9% | 5; 7.2% | χ^2^=24.32***, RR=7.21 | 12; 54.5% | 7; 9.5% | χ^2^=21.72***, RR=5.74 |
| ISI (M±SD) | 10.81±4.25 | 5.52±2.89 | t=7.20***, d=1.46 | 12.67±4.84 | 5.16±3.76 | t=8.09*** d=1.73 | 13.05±5.22 | 5.55±3.95 | t=7.26***, d=1.62 |
| PSQI (M±SD) | 6.81±2.81 | 3.63±1.91 | t=6.44***, d=1.32 | 7.00±2.90 | 3.75±1.95 | t=6.32*** d=1.32 | 6.73±2.90 | 4.06±2.30 | t=4.48***, d=1.02 |
| SL (M±SD) | 31.88±22.89 | 18.83±9.25 | t=3.97***, d=.75 | 32.96±24.62 | 19.35±9.31 | t=3.95*** d=.73 | 34.55±27.03 | 19.80±9.23 | t=4.01***, d=.73 |
| TST (M±SD) | 6.64±1.28 | 7.39±1.09 | t=-2.99**, d=.63 | 6.61±1.28 | 7.35±1.12 | t=-2.79** d=.62 | 6.55±1.39 | 7.32±1.09 | t=-2.73**, d=.62 |
| TST ≤ 6 (n;%) | 16; 50.0% | 8; 12.5% | χ^2^=16.00***, RR=4.00 | 13; 48.1% | 11; 15.9% | χ^2^=10.74**, RR=3.03 | 12; 54.5% | 12; 16.2% | χ^2^=13.29*, RR=3.36 |
| ESS (M±SD) | 10.81±4.25 | 5.52±2.89 | t=7.20***, d=1.46 | 11.26±3.89 | 5.72±3.20 | t=7.16***, d=1.56 | 11.55±4.07 | 6.01±3.35 | t=6.47***, d=1.49 |
| EPDS (M±SD) | 8.34±4.52 | 3.94±3.77 | t=5.05***, d=1.06 | 9.37±4.08 | 3.86±3.68 | t=6.41, p<.001, d=1.42 | 9.32±4.39 | 4.24±3.88 | t=5.22***, d=1.23 |
| EPDS ≥ 10 (n;%) | 13; 40.6% | 4; 6.3% | χ^2^=17.30***, RR=6.44 | 13; 48.1% | 4; 5.8% | χ^2^=23.89***, RR=8.29 | 11; 50.0% | 6; 8.1% | χ^2^=20.42***, RR=6.17 |
| EPDS ≥ 13 (n;%) | 8; 25.0% | 2; 3.1% | χ^2^=10.94***, RR=8.06 | 8; 29.6% | 2; 2.9% | χ^2^=14.86***, RR=10.21 | 7; 31.8% | 3; 4.1% | χ^2^=14.01***, RR=7.76 |
| SI (n;%) | 4; 12.5% | 2; 3.1% | χ^2^=3.20***, p=.074 | 4; 14.8% | 2; 2.9% | χ^2^=4.70***, RR=5.10 | 4; 18.2% | 2; 2.7% | χ^2^=6.94**, RR=6.74 |
| PFR (M±SD) | 2.84±1.17 | 1.89±.93 | t=4.35***, d=.90 | 3.04±1.16 | 1.88±.90 | t=5.19***, d=1.44 | 3.05±1.09 | 1.96±.99 | t=4.43***, d=1.05 |
| PSASC (M±SD) | 20.50±6.99 | 12.89±4.39 | t=6.52***, d=1.30 | 21.48±6.99 | 13.06±4.40 | t=7.07***, d=1.12 | 21.55±7.23 | 13.61±4.96 | t=5.89***, d=1.28 |

Note: n = group size. % = percentage of group. M±SD = mean and standard deviation. t = t-statistic for independent samples t-test. d = Cohen’s d effect size. χ^2^ = chi-square statistic. RR = risk ratio. p = significance value for non-significant result. * = p<.05. ** = p<.01. *** = p<.001. PSASC = pre-sleep arousal scale cognitive factor. PSASS = pre-sleep arousal scale somatic factor. Poverty = annual reported household income ≤ $20,000. ISI = insomnia severity index. PSQI = Pittsburgh sleep quality index. SL = sleep latency in minutes. TST = total sleep time in hours. TST ≤ 6 hours indicates short sleep. ESS = Epworth sleepiness scale. EPDS = Edinburgh postnatal depression scale. EPDS ≥ 10 detects minor and major depression. EPDS ≥ 13 indicates major depression. SI = suicidal ideation per the EPDS #10; a binary variable wherein any level of endorsement indicates a positive screen. PFR = perinatal focused rumination as measured via an appended item to the PSASC.

*Excessive sleepiness in prenatal insomnia?*

An incidental finding—unrelated to the study goals but nevertheless deserving of emphasis—is that two out of every three women with DSM-5 insomnia disorder reported excessive daytime sleepiness. Moreover, this was a large effect wherein pregnant women with insomnia reported rates 4x higher than those without insomnia. As insomnia is considered a 24-hour arousal disorder reflected in part by *lower* levels of sleepiness during the day (assessed objectively^1-3^ and subjectively^4, 5^) in the broader insomnia population, our data suggest that daytime sleepiness may be a unique and important daytime impairment in prenatal insomnia. Yet, we must be cautious in our interpretation.

Research on the relationship between insomnia/sleep disturbance and daytime sleepiness in perinatal women has yielded mixed findings, including both positive associations^6, 7^ and null results.^8, 9^ Review of these studies and our own did not immediately reveal that disparate findings were attributable to sample differences regarding gestational age or sociodemographic domains. Perhaps relevant here, however, is that distinctions between fatigue and sleepiness in the perinatal period are blurred and unclear as experienced by pregnant women.^7^ In contrast, fatigue and sleepiness are quite distinct, even if related, constructs in the non-perinatal population.^3^ As fatigue is a well-documented sequela of insomnia, the indistinction between fatigue and sleepiness in peripartum may contribute to the elevated rates of patient-reported daytime sleepiness in pregnant women with insomnia. Future studies investigating daytime sleepiness in pregnancy should utilize objective assays (e.g., multiple sleep latency test) to determine whether pregnant women with insomnia have greater objective daytime sleepiness than good sleeping pregnant women. If objective data replicate the present study findings, then daytime sleepiness would appear to play an important role in prenatal insomnia presentation. However, if objective data do not indicate that insomnia is associated with daytime sleepiness in pregnancy, then we may consider that the patient-reported sleepiness ratings may largely reflect fatigue experiences.

Ultimately, if daytime sleepiness has a role in prenatal insomnia, it could serve as consequence, contributor, or both. As a contributor to insomnia, daytime naps to combat daytime sleepiness can have unintended consequences on sleep drive and nighttime wakefulness. As a consequence, nocturnal wakefulness at night may directly increase daytime sleepiness. In addition, it is possible that daytime sleepiness affects maternal *perceptions* of sleep (e.g., *I am sleepy during the day, therefore I must not have slept well last night*). Longitudinal research is needed to clarify the role (if any) that daytime sleepiness plays in prenatal insomnia. If sleepiness is a common feature of prenatal insomnia in a notable segment of the patient population, that may have profound implications for insomnia intervention.

References

1. Roehrs TA, Randall S, Harris E, Maan R, Roth T. MSLT in primary insomnia: stability and relation to nocturnal sleep. *Sleep*. 2011;34(12):1647-1652.

2. Stepanski E, Zorick F, Roehrs T, Young D, Roth T. Daytime alertness in patients with chronic insomnia compared with asymptomatic control subjects. *Sleep*. 1988;11(1):54-60.

3. Riedel BW, Lichstein KL. Insomnia and daytime functioning. *Sleep medicine reviews*. 2000;4(3):277-298.

4. Pallesen S, Bjorvatn B, Nordhus IH, Sivertsen B, Hjørnevik M, Morin CM. A new scale for measuring insomnia: the Bergen Insomnia Scale. *Perceptual and motor skills*. 2008;107(3):691-706.

5. Cho YW, Song ML, Morin CM. Validation of a Korean version of the insomnia severity index. *Journal of clinical neurology*. 2014;10(3):210-215.

6. Wang W-J, Hou C-L, Jiang Y-P, et al. Prevalence and associated risk factors of insomnia among pregnant women in China. *Comprehensive psychiatry*. 2020;98:152168.

7. Insana SP, Montgomery-Downs HE. Maternal postpartum sleepiness and fatigue: Associations with objectively measured sleep variables. *Journal of psychosomatic research*. 2010;69(5):467-473.

8. Fernández-Alonso AM, Trabalón-Pastor M, Chedraui P, Pérez-López FR. Factors related to insomnia and sleepiness in the late third trimester of pregnancy. *Archives of gynecology and obstetrics*. 2012;286(1):55-61.

9. Wołyńczyk-Gmaj D, Różańska-Walędziak A, Ziemka S, et al. Insomnia in pregnancy is associated with depressive symptoms and eating at night. *Journal of Clinical Sleep Medicine*. 2017;13(10):1171-1176.
